# Simultaneous Oral and Umbilical Locations as a First Sign of Pemphigus Vulgaris

**DOI:** 10.1155/2021/7792360

**Published:** 2021-10-25

**Authors:** Eya Moussaoui, Yassine Oueslati, Lamia Oualha, Mohamed Denguezli, Badreddine Sriha, Nabiha Douki

**Affiliations:** ^1^Department of Dental Medicine, Sahloul Hospital (Sousse), Dental Faculty of Monastir, University of Monastir, Tunisia; ^2^Laboratory of Oral Health and Maxillofacial Rehabilitation (LR12ES11), University of Monastir, Tunisia; ^3^Dermatology Department, Farhat Hached Hospital (Sousse), University of Sousse, Tunisia; ^4^Cytology and Pathological Anatomy Department, Farhat Hached Hospital (Sousse), University of Sousse, Tunisia

## Abstract

The place of pemphigus vulgaris (PV) among autoimmune bullous diseases (AIBD) is well established. It is an acquired chronic, autoimmune, vesiculobullous disease in which IgG antibodies target desmosomal proteins to produce intraepithelial mucocutaneous blistering. The diagnosis is often challenging for the clinicians. It requires a combination of three major features: clinical, histopathological, and immunological. Clinically, oral lesions are the first manifestations of the disease in 50-90% of the patients with widespread blisters affecting the oral mucosa. On the skin, lesions are characterized by flaccid blisters that rapidly progress into erosions and crust formation. Umbilical lesions as a clinical manifestation of PV are peculiar and have rarely been reported, and they are not yet completely elucidated. Umbilical region involvement in patients with pemphigus was assessed in a limited study totalling just 10 patients. This localisation may be a valuable hint easing the diagnosis at the clinical level for patients with oral mucosal blisters. Dentists must be familiar with the clinical manifestations of PV to make an early diagnosis and start an early treatment which determines the prognosis of the disease. To the best of the authors' knowledge, the coexistence of these lesions with the oral lesions as a first sign of PV in the absence of skin involvement was reported in only one case of pemphigus vegetans (PVe). In this paper, we describe an observation of a female patient that was diagnosed with PV that begun with simultaneous oral and umbilical locations which coexisted for a period of 4 months before the appearance of other cutaneous lesions. We highlight the role of dentists, by being familiar with the clinical manifestations of PV, to make an early diagnosis to start an early treatment which determines the prognosis of the disease and to follow closely the evolution of lesions to change treatment if required. We also discuss the clinical, histological, and immunological features of the disease that enabled the differential diagnosis as well as the appropriate therapeutic management.

## 1. Introduction

Autoimmune bullous diseases (AIBD) constitute a heterogeneous group of diseases that are at the same time very diverse, infrequent, and of variable prognosis, sometimes pejorative. The place of pemphigus vulgaris (PV) among this group is well established. It is an acquired chronic, autoimmune, vesiculobullous disease in which IgG antibodies target desmosomal proteins (Desmogleins 1 and 3) to produce intraepithelial mucocutaneous blistering [1]. It owes its name to the Greek word “pemphix” which means blister and was first used by Hippocrates in 460-370 B.C. [2]. It is a rare disease with an estimated worldwide annual incidence between 1 and 5 per million [3]. It occurs in all racial and ethnic groups with the highest incidence seen in Ashkenazi Jewish, Mediterranean, Indian, Malaysian, Chinese, and Japanese descent [4]. Occurrence is most common during the fifth and sixth decades of life, although a few cases have been reported in children [5].

Diagnosis of PV is often challenging for the clinicians. It requires a combination of three major features: clinical, histopathological, and immunological, which represent the gold standard for autoimmune blistering diseases [6]. Delay in diagnosis affects the prognosis of the disease and increases the risk of infection and duration of hospitalization and treatment, in addition to reducing the patient's quality of life.

In this report, we describe an observation of a female patient that was diagnosed with PV that begun with simultaneous oral and umbilical locations which coexisted for a period of 4 months before the appearance of other cutaneous lesions. We highlight the role of dentists, by being familiar with the clinical manifestations of PV, to make an early diagnosis to start an early treatment which determines the prognosis of the disease and to follow closely the evolution of lesions to change treatment if required.

We also discuss the clinical, histological, and immunological features of the disease that enabled the differential diagnosis as well as the appropriate therapeutic management.

## 2. Case Presentation

**Figure 1:**
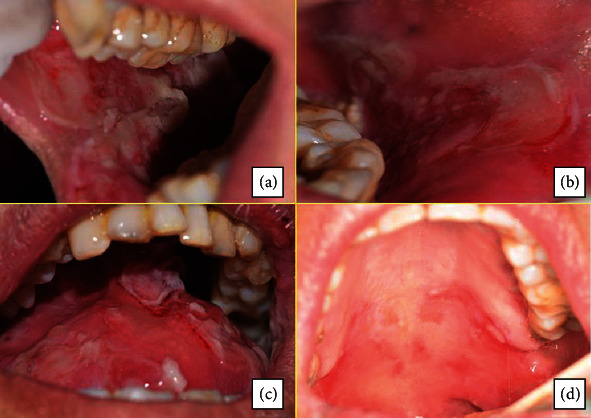
Mucosal damage affecting the entire oral cavity. The lesions were located in the internal surfaces of the lips and cheeks, the dorsal surface of the tongue, and the palatal fibromucosa. There were multiple epithelial erosions and postbullous ulcerations.

**Figure 2:**
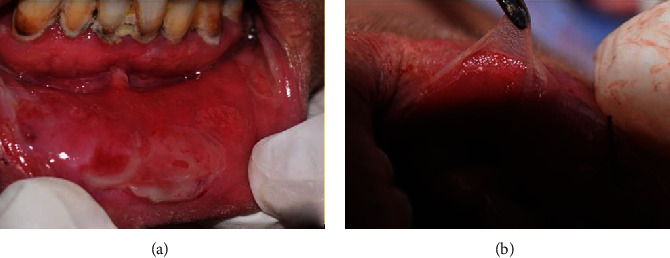
Blisters of the oral mucosa with a positive Nikolsky sign. Presence of poor oral hygiene-associated bullous lesions and ulcerations. Nikolsky sign: epithelial cells are separated from the basement membrane, allowing an assessment of the fragility of epithelial attachment mechanisms (acantholysis).

**Figure 3:**
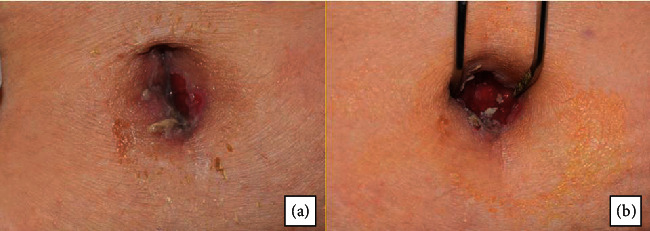
Aspect of the umbilical lesions. Erosions and ulcerations with erythema affecting the umbilical area associated with Staphylococcus aureus growth.

A 55-year-old woman with no notable medical and family history was referred to our department by her general practitioner following the appearance of multiple lesions of the oral mucosa. The intraoral clinical examination revealed poor oral hygiene, hypersialorrhea, and extensive mucosal damage affecting the entire oral cavity and causing discomfort, pain, and dysphagia. The lesions were located in the internal surfaces of the lips and cheeks, the dorsal and ventral surface of the tongue, and the soft palate mucosa (Figure 1). There were epithelial erosions and postbullous ulcerations with a positive Nikolsky sign indicating acantholysis with dislocation of the epithelial layers (Figure 2). Bulloerosive umbilical involvement was associated with the oral impairment without noting any other cutaneous lesions for four months (Figure 3).

**Figure 4:**
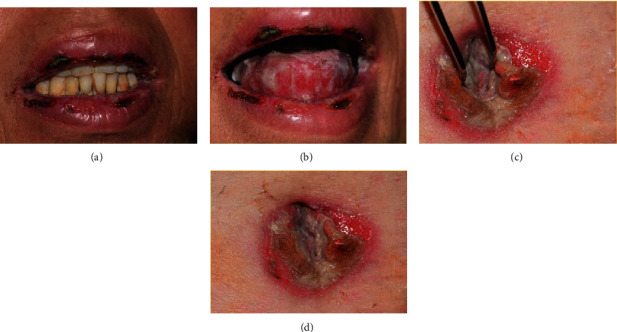
Worsening of the lesions 5 days after the first consultation. Extension of the lesions with appearance of multiple crusts in the lips and umbilicus.

In front of these signs, we conducted an oral biopsy and prescribed topical prednisolone (20 mg; 3 times per day) and chlorhexidine-based (0.12%; 3 times per day) mouthwashes in order to reduce the oral pain awaiting the biopsy result. In a while, her condition worsened (Figure 4). We referred her to the department of dermatology where she was hospitalized and an umbilical biopsy was performed.

**Figure 5:**
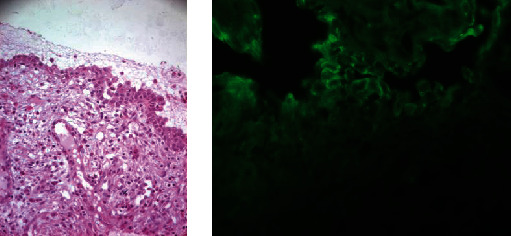
Histopathological and IFD of the oral biopsy. The oral biopsy concerned a squamous mucosa lined with a partially visualized coating, with disappearance of the superficial layers and the presence of cells of the basal layer which remain attached to the underlying chorion, drawing a “tapestry nail” appearance, surmounted by a few acantholytic cells. In the chorion, we noted the presence of a polymorphic inflammatory infiltrate rich in eosinophilic polynuclear cells. Direct immunofluorescent examination was negative.

**Figure 6:**
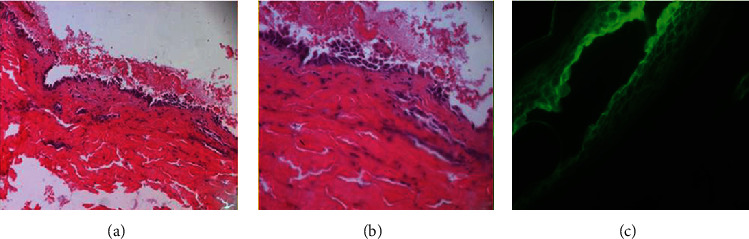
Histopathological and IFD of the skin biopsy. The sample received was carried to the deep dermis. It was bordered by the basal layer of the epidermis, drawing the same “tapestry nail” appearance with extensive suprabasal acantholysis and separation of the overlying epidermal layers. The dermis was the site of an inflammatory infiltrate rich in neutrophils and eosinophils of perivascular arrangement with extravasation of red blood cells and without alteration of their wall. Intercellular immunoglobulin G and C3 deposits were observed on direct immunofluorescent examination with a characteristic mesh-like appearance.

On histopathological examinations, the oral biopsy concerned a squamous mucosa lined with a partially visualized coating, with disappearance of the superficial layers and the presence of cells of the basal layer which remain attached to the underlying chorion, drawing a “tapestry nail” appearance, surmounted by a few acantholytic cells. In the chorion, we noted the presence of a polymorphic inflammatory infiltrate rich in eosinophilic polynuclear cells. Direct immunofluorescent examination was negative (Figure 5). As for the umbilical biopsy, the sample received was carried to the deep dermis. It was bordered by the basal layer of the epidermis, drawing the same “tapestry nail” appearance with extensive suprabasal acantholysis and separation of the overlying epidermal layers. The dermis was the site of an inflammatory infiltrate rich in neutrophils and eosinophils of perivascular arrangement with extravasation of red blood cells and without alteration of their wall. Intercellular immunoglobulin G and C3 deposits were observed on direct immunofluorescent examination with a characteristic mesh-like appearance (Figure 6). Based on clinical, histopathological, and immunofluorescence findings, a diagnosis of pemphigus vulgaris (PV) was established.

On laboratory investigations, we found an inflammatory syndrome with increased erythrocyte sedimentation rate (ESR) and C-reactive protein (CRP). Hepatitis (VHB and VHC) and VIH serologies were negative. Mycological direct examination and cultures were negative. On bacteriological culture performed from the umbilicus, *Staphylococcus aureus* growth was observed.

As for the therapeutic management, due to oral pain, a nasoenteral probe was used to feed her. Daily intravenous administration of prednisolone (120 mg/day) and amoxicillin-clavulanic acid (1000 mg thrice a day) was started. The nursing team was instructed to clean the oral cavity and the umbilicus using chlorhexidine (0.12%) mouthwash and ointment. Her condition improved gradually during hospitalization. There was a complete remission of the lesions in the mouth and umbilicus. The patient was advised to continue oral prednisone intake at a dose of 20 mg/day. She underwent monthly follow-ups in dentistry and dermatology departments after discharge from the hospital.

Four months later, the disease recurred. We noticed the appearance of severe cutaneous lesions added to the oral and umbilical ones located in the elbows, forearms, and the periungual area. She was newly hospitalized.

## 3. Discussion

This report describes a case of pemphigus vulgaris (PV) beginning with simultaneous oral and umbilical locations coexisting for a period of 4 months before the appearance of other cutaneous lesions. Umbilical lesion as a clinical manifestation of pemphigus has been reported very rarely so far and was assessed in a limited study totaling just 10 patients [7]. The reasons why an umbilical lesion may be a presenting feature of pemphigus remain unclear [7].

Recognition of this presentation for patients with PV is relevant; in fact, clinicians generally recall blistering diseases in the navel area only in pregnant and lactating females [8]. It is known that pemphigoid gestationis blisters occur in the umbilical region and later spread to the rest of the body [9]. However, this localisation in mucocutaneous PV is unusual. A possible explanation may be related to antibodies binding to the cadherin superfamily in embryonic remains on the umbilical cord or placenta [7].

In a series of 1209 cases of pemphigus (1111 PV) with involvement of different regions, Chams-Davatchi et al. reported that initial localisation was solely oral in 62./. and umbilical in only 3./. [10]. Very few cases of pemphigus vegetans in the navel area were reported [11, 12]. The coexistence of oral and umbilical lesions as a first sign of PV in the absence of skin involvement was reported in only one case of pemphigus vegetans (PVe). The female patient was followed up for more than one year without appearance of other localisations [12]. For our patient, oral and umbilical lesions were the first sign of PV before the appearance of other cutaneous lesions after four months. Jałowska et al., in a series of 81 PV with nail apparatus involvement and navel area involvement, have found that only females had lesions in the navel area. This correlates with our case reported. Authors speculate therefore that a relationship may exist between the pattern of expression of PV lesions around the umbilicus and the female reproductive system [9], whereas Oliveira Junior et al., in a limited study of 10 patients having pemphigus with umbilical involvement (5 PV, 5 P. foliaceus), showed that between 5 patients with mucocutaneous PV, one was male. In the same series, umbilical lesions were, as in our case, crusts, erosions, and erythema [7].

Diagnosis of the PV is based on identification of clinical manifestations and confirmation by incisional biopsy of the affected perilesional tissue of the skin or mucous membranes [13]. Clinically, oral lesions are the first manifestations of the disease in 50-90% of the patients with widespread blisters affecting the oral mucosa, most commonly in areas subjected to frictional trauma [14]. On the skin, lesions are characterized by flaccid blisters that rapidly progress into erosions and crust formation and occasionally develop opportunistic infections [15]. At the end of the 19^th^ century, a Russian dermatologist, Pyotr Vasiliyevich Nikolsky, first described a clinical sign characterized by blisters spreading upon the application of a horizontal, tangential pressure to the skin and which would subsequently become widely known as “Nikolsky's sign.” When this sign tested positive, epithelial cells are separated either from one another or from the basement membrane, allowing an assessment of the fragility of epithelial attachment mechanisms [16].

These features were observed in our patient with extensive blisters affecting the oral mucosa, a positive Nikolsky sign and a bulloerosive umbilical involvement.

Oral mucosal blisters can recall many differential diagnoses making the clinical diagnosis difficult. They include other autoimmune bullous diseases (AIBD), erosive lichen planus, and *Stevens-Johnson* and *Lyell* syndromes, as well as the lesions caused by acute viral infections like *Herpes simplex* and erythema multiforme. Dentists must be familiar with the clinical manifestations of PV [14]. In fact, the oral cavity may be the only site of involvement for many months before the appearance of other localisations, and this may lead to delayed diagnosis and inappropriate treatment of a potentially fatal disorder [14]. These lesions may occur anywhere on the oral mucosa, but mostly in the buccal mucosa followed by palatal, lingual, and labial mucosa. The gingiva is the least commonly affected site, and the commonest manifestation is desquamative gingivitis. In this case, oral lesions were located only in nonkeratinized mucosa (internal surfaces of the lips and cheeks, the dorsal surface of the tongue, and the soft palate) [14]. The clinician should be able to distinguish the lesions of PV from those caused by other diseases [14]. For recurrent aphthous stomatitis, oral ulcers (aphthae) are surrounded by an erythematous halo and regular margins with yellowish base [17, 18]. Acute herpetic gingivostomatitis is associated to prodromic symptoms followed by the onset of small yellowish vesicles that rapidly rupture, giving rise to ulcers with an erythematous halo. It affects mostly keratinized mucosa [18]. In cicatricial pemphigoid, skin lesions precede oral lesions, and blisters are smaller with a shorter duration than in PV. Bullous pemphigus is rare on the mucosa and develops on normal or erythematous skin with intense pruritus and symmetric lesions that appear on flexion areas, root of extremities, thighs, and abdomen. For erythema multiform, the presence of target-shaped skin lesions will make the diagnosis. Erosive lesions in erosive lichen planus are charaterised by the presence of Wickham striae [17]. Lyell syndrome and Stevens-Johnson syndrome are severe dermatosis that are mostly caused by medication. They are characterized by toxic epidermal necrolysis in which epidermal detachment affects more than 30% of the body surface area in Lyell syndrome but affecting less than 10% of the body surface area in Stevens-Johnson syndrome. A positive Nikolsky sign and a positive Asboe Hansen sign are confirmatory signs. In these dermatosis, skin involvement usually occurs prior to mucous membrane involvement and the rapid withdrawal of the drug improves prognosis [19, 20].

The association of only oral and umbilical erosive postbullous lesions was not mentioned in the literature with the previous diseases, so it may be a valuable hint easing the diagnosis at the clinical level and recalling PV in patients other than pregnant and lactating females [9].

As for the histopathological features, the oral and the skin biopsy shared the same features with extensive suprabasal acantholysis affecting the epithelium and a “tapestry nail” appearance. As for the direct immunofluorescence, it was negative in the oral biopsy and positive in the skin biopsy. This result is possibly due to the fact that the oral biopsy was carried in an ulcerated area which affected the final result. In fact, blisters in the oral mucosa are short-lived; they have a very thin roof that could be rapidly ulcerated because of the presence of teeth [17]. It is recommended that the incisional biopsy should be done in a perilesional intact or clinically uninvolved tissue of the skin or mucous membranes [16]. In our case, the diagnosis was confirmed by the characteristic deposition of IgG and C3 antibodies that bind to cell surface painting a mesh-like appearance of the umbilical lesion. The navel area involvement can therefore facilitate the performance of DIF in individuals with very painful or seriously damaged oral lesions.

Therapeutic management requires a multidisciplinary approach. Patients with PV need to maintain good oral hygiene, which may be compromised due to pain and blistering. Oral conditions can worsen with an accumulation of dental biofilm, causing gingivitis, or exacerbation of preexisting periodontal disease. Daily use of topical corticosteroids (prednisolone) and chlorhexidine mouthwashes helps to reduce pain and inflammation. As for systemic treatment, corticosteroids are most effective to control disease activity either alone or in combination with other immunosuppressive/immunomodulant drugs such as azathioprine, mycophenolate, and rituximab [21]. Our patient showed a gradual improvement in her condition after systemic corticosteroid intake, but PV lesions rapidly recurred, worsened, and expanded to other skin lesions requiring the addition of immunosuppressive therapy. Apart from the blisters, the risk of infection is always present. We treated a coexisting infection (*Staphylococcus aureus*) with amoxicillin-acid clavulanic.

Before the advent of corticosteroid therapy, pemphigus was fatal, with a mortality rate of up to 75% in the early 1950s [22]. It is still a life-threatening disorder. For every patient, the optimal management requires 3 crucial steps: an accurate diagnosis, a correct evaluation of the spread and severity of the disease, and a comprehensive analysis of his systemic condition (age, comorbidities) and, if present, the side effects of previous therapies [15]. Multidisciplinary teams are extremely important for providing comprehensive patient care, preventing further damage, improving the prognosis and quality of life, and reducing the length of hospital stay. Added to that, regular monitoring is the major rule to detect any recurrence of the disease.

## 4. Conclusion

This report described the case of a female patient that was diagnosed with mucocutaneous PV which begun with simultaneous oral and umbilical locations coexisting for a period of 4 months before the appearance of other cutaneous lesions. The association of these two localisations may be a valuable hint easing the diagnosis at the clinical level for patients with oral mucosal blisters. Further reports and studies are necessary to achieve a better understanding of the disease in order to give the appropriate care.
